# Active aging and health among older adults in China: a perspective based on downward intergenerational economic support

**DOI:** 10.3389/fpubh.2024.1337829

**Published:** 2024-06-17

**Authors:** Liu Yuanfeng, Zhang Xu

**Affiliations:** College of Public Administration and Law, Hunan Agricultural University, Changsha, Hunan, China

**Keywords:** downward intergenerational economic support, health of older adults, mediating effects, instrumental variable models, cross-sectional threshold models

## Abstract

**Introduction:**

In China, the rapid progression of population aging presents significant challenges to society and the economy, drawing widespread attention to the health conditions of older adults. While aging is often seen as a societal burden, the phenomenon of intergenerational economic support reveals the potential for older adults to continue playing an active role within their families. This study delves into how older parents’ financial support to their children can reciprocally influence their own health, exploring the potential non-linear relationships involved.

**Methods:**

This research, utilizing data from the 2018 China Health and Retirement Longitudinal Study, employs instrumental variable techniques and cross-sectional threshold models to examine how financial support provided by older adults to their children affects their health. It particularly highlights the varied impacts of economic support on older adults’ health at different levels of support.

**Results:**

The findings indicate that moderate intergenerational economic support significantly enhances the health of older adults, while either minimal or excessive financial support does not demonstrate the same positive effect. Additionally, subjective life expectancy plays a mediating role between intergenerational economic support and the health of older adults, further emphasizing the beneficial impact of economic support.

**Discussion:**

The study underscores the importance of moderate intergenerational economic support in improving the health of older adults amidst aging challenges. Future policies and practices should consider how to encourage and optimize such support to address the challenges of an aging society, enhance the welfare of older adults, and promote healthy aging.

## 1 Introduction

The 21st century is widely recognized as the age of aging, a phenomenon driven by significant declines in fertility rates and extensions in life expectancy. This demographic shift has triggered concern for the health and well-being of older populations globally, especially in countries experiencing rapid aging. The World Health Organization’s report on “Aging and Health” notes that by 2020, the global population aged 60 years and older surpassed the number of children under 5 years. By 2030, one-sixth of the world’s population will be over 60, and by 2050, this figure is expected to rise to 2.1 billion. Notably, aging is no longer a characteristic unique to developed nations like Japan; by 2025, 80% of the older population will reside in low and middle-income countries (1). Specifically, China, the world’s largest developing country, faces significant aging challenges. As of the end of 2022, the population aged 60 years and older in China was 280.04 million, accounting for 19.8% of the total population; those 65 and older accounted for 14.9%. The proportion of the population aged 60 and over in China increased by 0.9%, and those 65 and over by 0.7% compared to 2021, highlighting an intensifying trend of population aging (2). Concurrently, the health status of China’s older adults is less than optimal, with over 190 million people aged 60 and older suffering from chronic diseases such as obesity, hypertension, and diabetes in 2022 (3, 4). As China’s older population grows, improving their health status is crucial for advancing healthy aging and effectively responding to the national strategy against aging.

The World Health Organization defines healthy aging as maintaining and enhancing an individual’s functional ability during old age, enabling them to stay healthy, participate in social life, and maintain independence and autonomy. This definition underscores the active role of older adults in society and families, noting that their health is not merely a matter of physical wellness but also their capacity to interact socially and achieve personal value. Simultaneously, the Chinese government has increasingly emphasized the promotion of a positive view on aging and the concept of healthy aging, implementing a series of policies and measures such as the “Opinions of the CPC Central Committee and the State Council on Strengthening Aging Work in the New Era” and the “National Plan for the Development of Aging Affairs and Elderly Care Service System during the 14th Five-Year Plan.” These policies aim to tap into the potential of an aging society, invigorate its dynamics, and promote the healthy aging of older adults. Against this backdrop, the concept of intergenerational support gains more importance. Intergenerational support involves the reciprocal exchange of economic, life, and emotional assistance between generations within a family, sharing life experiences and resources (5). Such support is not limited to one-way assistance from children to older parents; older adults continue to support their children’s daily lives in various ways, such as financing their children’s home purchases or car buys, or caregiving for grandchildren. According to surveys, a significant portion of older adults engage in “financial recirculation” to their children. The survey data shows that 52.4% of older adults spend more on their children, primarily through financial support for home and vehicle purchases, accounting for 29.3 and 12.7%, respectively. Additionally, 43.5% of older adults also make purchases for their grandchildren, indirectly supporting their children financially (6).

Despite widespread attention to aging and its implications for individuals and society, current research lacks depth on how intergenerational support affects the health of older adults. Economic support, a common form of mutual aid in Chinese families, significantly influences the health and well-being of older adults. However, academic understanding of how older adults’ downward economic support impacts their own health remains limited. This research gap is particularly critical in rapidly aging societies like China, where families play a central role in providing health and social support for older adults. This study aims to explore the relationship between intergenerational economic support provided by older adults and their health status, seeking to fill the gaps in existing literature and provide a scientific basis for policy-making.

## 2 Literature review and theoretical analysis

### 2.1 Literature review

Health is fundamental to ensuring that older adults enjoy their later years independently and autonomously. Enhancing the health levels of older adults is a critical task in building a healthy China and a proactive response to the challenges of an aging population. Since the late 1980s, the concept of health has expanded beyond mere physiological indices such as sickness and mortality rates to include a comprehensive concept encompassing physical, psychological, and social health. This expansion has complicated the construction of health indicators for older adults, leading to diverse opinions among scholars. For instance, Peter and Lorraine, among other scholars, use self-rated health to represent physical health status (7, 8), though this measure alone introduces a degree of subjective heterogeneity; Zhang divides the health of the older adults into three categories: physical health, cognitive function, and self-rated health (9); Okamoto assesses the intrinsic capabilities of older adults by evaluating challenges in basic and instrumental activities of daily living (ADL and IADL) and cognitive functions, considering also the certification status for public long-term care needs (10); Ye adopts the presence of chronic diseases as a measure of physiological health and evaluates the psychological health of the older adults through cognitive issues and depression symptoms (11); Lv and Zhang measure the physical health of older adults from both subjective and objective perspectives, using indicators such as receiving medical treatment and hospitalization (12); Yao and colleagues use the EQ-5D-3L scale’s five dimensions as standards to assess individual health status, including mobility, self-care, usual activities, pain/discomfort, and anxiety/depression, utilizing scores from the Visual Analog Scale (VAS) and utility values U to quantify quality of life (13).

Intergenerational support represents a bidirectional flow of resources between two generations within a family, encompassing economic support, caregiving, and emotional exchange (14). Extensive research has focused on the relationship between intergenerational support and health, predominantly examining support provided by children to older adults. Several studies have identified a positive correlation between upward intergenerational support and health. For example, Shu and colleagues, using structural equation modeling, found that upward intergenerational support from children can significantly improve the health of parents, especially their psychological well-being (15); Li and Guo, employing Heckman selection models and IV-Probit models, demonstrated that economic and emotional support from children can enhance parents’ capabilities in Activities of Daily Living and Instrumental Activities of Daily Living (16); Xu argues that living with children benefits the psychological health of older adults, and economic and emotional support from children has a significant positive impact on their psychological health (17). Qin’s analysis using logistic regression models indicates that older adults who provide economic support have lower rates of poor self-rated health (18). Conversely, some studies suggest that excessive support from children can reduce older adults’ sense of self-efficacy, leading to negative attitudes toward aging and diminishing psychological health (19).

While research on the health impacts of older adults’ giving behaviors is less common, some scholars have examined intergenerational support from the perspective of older adults providing for their descendants. Thomas, using ordinary least squares regression, explored the impact of the type of support (given and received) on well-being, finding that providing support benefits older adults’ welfare more than support received from spouses or siblings, including support provided to children (20); Peng and colleagues, through multivariate linear regression, discovered that older adults who financially support their children gain more self-esteem, thereby enhancing their well-being and improving their health conditions (21). However, Schwarz argues that the giving behaviors of older adults can also have adverse effects on their psychological health if elders continually assist younger generations, which may be perceived as a failure in parental education, thus placing psychological pressure on older adults (22).

In summary, while previous studies provide a robust foundation for exploring the relationship between intergenerational support and the health of older adults, there is still room for improvement and addition. Although the relationship between intergenerational support and the health or welfare of older adults has been extensively discussed, research on downward intergenerational support remains insufficient. The academic consensus on whether intergenerational support positively impacts older adults’ health is not yet unified, with most empirical studies examining linear relationships between economic support and health, while few consider potential nonlinear relationships. Moreover, many studies examine health from various dimensions but typically analyze the relationship between intergenerational support and older adults’ health from a singular health perspective, especially psychological health. Thus, this paper will analyze the relationship between downward intergenerational economic support and the comprehensive health status of older adults from the perspective of downward intergenerational economic support, utilizing instrumental variable methods to identify the impact of intergenerational economic support on older adults’ health status. To explore the nonlinear relationship between intergenerational economic support and older adults’ health, threshold regression models will be further employed to investigate the threshold effects of downward intergenerational economic support on older adults’ health, aiming to explore pathways and challenges to enhancing the quality of life and living standards of older adults, thereby providing theoretical reference and practical guidance for relevant economic and social decisions.

### 2.2 Theoretical analysis

#### 2.2.1 Intergenerational economic support and older adults’ health

Within the framework of family economics, individuals often exhibit altruism and a preference for family coherence, incorporating the needs of other family members into their labor supply decisions (23). Influenced by the ethics of family-centric responsibilities, parents often provide for their children and family selflessly, resulting in a downward allocation of resources within the family (24). In China, most older adults rely on themselves as much as their health permits and provide financial support to their children to alleviate their living pressures. This willing provision of support, sometimes humorously referred to as being ‘nibbled on’ by one’s own children, is seen by many older adults as a fulfilling part of parental duties. Witnessing their children’s improved living conditions due to their support not only brings older adults a sense of achievement and satisfaction but also positively impacts their mental and physical health.

#### 2.2.2 Intergenerational economic support, subjective life expectancy, and older adults’ health

Subjective life expectancy reflects older adults’ unique perception of their aging process, embedding these perceptions into their self-concept and identity (25). The arrangements and plans people make for their future lives are influenced by their expected lifespan. Providing economic support to children implies that older adults need to work and have their own sources of income. Activity theory suggests that maintaining social activity can slow the aging process (26), fostering a positive view of aging among older adults. The process of earning an income independently provides older adults with a sense of self-worth. Consequently, intergenerational economic support can positively affect older adults’ views on aging and enhance their subjective life expectancy. A positive attitude toward aging can significantly reduce the occurrence of psychological issues such as depression, thus improving older adults’ health levels.

## 3 Models and methods

### 3.1 Baseline regression model

Building on the analysis presented, this study constructs a baseline regression model to empirically test the impact of intergenerational economic support on the health status of older adults:


(1) $${\text{Health}}_{i}=\beta_{0}+\beta_{1}Gene_{i}+\beta_{2}{\text{Controls}}_{i}+\varepsilon_{i}$$

In Equation (1), ${\text{Health}}_{i}$ represents the overall health status of older adults; $Gene_{i}$ denotes intergenerational economic support; ${\text{Controls}}_{i}$ includes a series of control variables, such as the gender, age, income, number of living children, number of grandchildren, and satisfaction with child relationships of the surveyed older adults; i indexes the sample; $\beta_{0}$ is the constant term; $\beta_{1}$ & $\beta_{2}$ represent the estimated coefficients; ε is the random error term.

### 3.2 Threshold regression model

Traditional linear regression methods do not address structural breaks. The impact of intergenerational economic support on older adults’ health may vary with the level of financial support provided to their children, exhibiting distinct characteristics. Therefore, this study employs the threshold model proposed by Hansen (27) to further examine how intergenerational economic support affects older adults’ health across different support intervals. The model is expressed as follows:


(2) $$\begin{array}{l} {\text{Health}}_{i}=\delta_{i}+\alpha_{1}Gene_{i}I\left(Gene_{i}\leq \varphi \right)+\alpha_{2}Gene_{i}I\left(Gene_{i}>\varphi \right)\\ \;+\alpha_{n}{\text{Controls}}_{i}+\sigma_{i} \end{array}$$

In Equation 2, ${\text{Health}}_{i}$, $Gene_{i}$, and ${\text{Controls}}_{i}$ have the same meanings as in Equation 1; I(·) represents the indicator function; $\delta_{i}$ is the constant term; $\alpha_{1}$,$\alpha_{2}$,$\alpha_{n}$ are coefficients of the influencing factors; $\sigma_{i}$ is the random error term.

### 3.3 Mediation effect model

To more finely explore the mechanisms through which intergenerational economic support impacts older adults’ health, based on the theoretical analysis, a stepwise regression method (28) is used to validate the mediation effect model, set up as follows:


(3) $$He{\text{alth}}_{i}=\beta_{0}+\beta_{1}Gene_{i}+\beta_{2}{\text{Controls}}_{i}+\varepsilon_{i}$$

(4) $$Life_{i}=\theta_{0}+\theta_{1}Gene_{i}+\theta_{2}{\text{Controls}}_{i}+\partial_{i}$$

(5) $${\text{Health}}_{i}=\epsilon_{0}+\epsilon_{1}Gene_{i}+\epsilon_{2}Life_{i}+\epsilon_{3}{\text{Controls}}_{i}+\omega_{i}$$

Equation 3 tests the impact of intergenerational economic support on older adults’ health; Equation 4 examines the influence of intergenerational economic support on subjective life expectancy; and Equation 5 tests the mediation effect of subjective life expectancy. Here, ${\text{Health}}_{i}$ represents older adults’ health; $Gene_{i}$ is intergenerational economic support; $Life_{i}$ is the subjective life expectancy of older adults; ${\text{Controls}}_{i}$ represents the control variables; $\beta_{0}$,$\theta_{0}$,$\epsilon_{0}$ are constant terms; $\beta_{1},\beta_{2},\theta_{1},\theta_{2},\epsilon_{1},\epsilon_{2},\epsilon_{3}$ are coefficients of the influencing factors; $\varepsilon_{i},\partial_{i},\omega_{i}$ are random error terms.

## 4 Data source, variable selection, and descriptive statistic

### 4.1 Data source

This study utilizes data from the China Health and Retirement Longitudinal Study (CHARLS), which is a long-term project managed by the National Development Research Institute of Peking University. The CHARLS project aims to collect longitudinal data on the health, retirement, and economic status of the Chinese population aged 45 and above. The sampling design of CHARLS considers the representativeness of the national population, employing Probability Proportional to Size Sampling (PPSS) and Computer Assisted Personal Interviewing (CAPI) techniques to randomly select multi-stage samples (county/district-village/community-household), ensuring comprehensive coverage (29). The project team has implemented high-standard training and strict data quality control measures to ensure consistency and accuracy in data collection. The design and implementation framework of CHARLS, detailed in the cohort profile by Zhao et al. published in the International Journal of Epidemiology, validates the reliability and validity of the survey (30). The data source relied upon in this study demonstrates high credibility and validity, providing a solid foundation for our analytical results.

The 2018 questionnaire included sections on family information, health status, cognition and depression, healthcare and insurance, work and retirement, asset income, and property, among others. Based on these considerations, the data is deemed to align well with the objectives of this paper, namely, the relationship between intergenerational economic support and the health of older adults. Therefore, according to the definitions of older adults by global organizations and the Chinese government, specifically those aged 60 and above (31), this study selects samples of individuals aged 60 or older who have children. Due to the CHARLS questionnaire allowing respondents to opt for “refuse to answer” or “do not know,” samples missing key variables such as economic support and health were excluded, ultimately retaining a total of 5,137 sample data points.

### 4.2 Variable definition and descriptive statistics

(1) Dependent Variable: Overall Health of Older Adults. This study measures the health of older adults using both subjective and objective indicators. This includes self-assessed health status (“How would you rate your health?”), physical health (including “suffering from pain” and “presence of chronic diseases”), and mental health indicators derived from an epidemiological survey on depression, such as “bothered by trivial things,” “difficulty concentrating,” “feeling down,” “everything feels like an effort,” “feeling fearful,” “sleeping poorly,” “feeling lonely,” and “feeling unable to carry on with life.” A measurement model is used to compute the overall health status of the respondents.
(2) Explanatory Variable: Intergenerational Economic Support. This is determined by asking older adults about the amount of economic support (both monetary and in-kind) they have provided to their children over the past year.
(3) Instrumental Variables. The study explores the causal relationship between intergenerational economic support provided by older adults to their children and their health status. Given the potential strong endogeneity between intergenerational economic support and health—where healthier older adults may be able to work longer and provide more support, and conversely, where various uncontrolled factors could affect health—this study uses instrumental variables for estimation. Education level and marital status are chosen as instrumental variables. Education, as a socio-economic resource, influences a person’s socio-economic status, thereby affecting intergenerational economic support. Additionally, spousal agreement can significantly influence the decision to provide financial support to children. However, education and marital status do not directly impact older adults’ health, thus theoretically validating the choice of these instrumental variables.
(4) Mediating Variable: Subjective Life Expectancy. Subjective Life Expectancy. Based on the theoretical analysis discussed previously, this paper selects subjective life expectancy as the mediating variable. The questionnaire assesses whether respondents believe they can live to specific ages, which correspond to their current age group: individuals aged 60–65 are asked if they expect to live to 75, those aged 65–69 to 80, those aged 70–74 to 85, those aged 75–79 to 90, those aged 80–84 to 95, those aged 85–89 to 100, those aged 90–94 to 105, those aged 95–99 to 110, and those over 100 to 115.
(5) Control Variables. Reflecting individual characteristics and family dynamics, the study includes sex, age, income, number of living children, number of grandchildren, co-residence with children, and satisfaction with child relationships as control variables. Table 1 presents the definitions of these variables along with descriptive statistics.

**Table 1:** Variable definitions and descriptive stats.

Variable type	Variable	Definition	Mean	Std. Dev.
Dependent	Overall health	Older adults health, mean = 0	−0.009	0.581
Independent	Intergenerational economic support	Total value (in thousands) given to children (monetary and goods)	3.248	10.061
Control	Gender	Female = 1; Male = 0	0.472	0.499
	Age	Respondent’s age (years)	68.285	6.192
	Income	Total income (wages and transfers) logged	7.327	3.051
	Living children	No. of living children	3.147	1.512
	Grandchildren	No. of grandchildren	4.593	3.148
	Co-residence with children	Co-residing with children = 1; No = 0	0.148	0.355
	Satisfaction with child relations	Satisfied = 1; Not satisfied = 0	0.959	0.263
Instrumental	Highest education	Illiterate = 1; Elementary = 2; Junior high = 3; High school = 4; College or above = 5	1.974	1.097
	Marital status	Co-residing with partner = 1; No = 0	0.841	0.366
Mediating	Subjective life expectancy	Possible = 1; Not possible = 0	0.623	0.485

According to the descriptive statistics shown in Table 1, the health status of older adults in the sample is uniformly distributed, indicating that there is a similar proportion of respondents with good health and poor health. In terms of downward intergenerational support, the data shows that, on average, older adults provide their children with financial assistance amounting to 3,248 yuan, reflecting the traditional Chinese family norm of intergenerational mutual aid. Notably, the proportion of older adults living with their children is relatively low, which may be attributed to changes in family structures in China and the mobility of younger generations. Marital status data further indicates that many older adults are either living alone or cohabiting with their spouses. The expected subjective lifespan is also relatively high, showcasing the prevailing retirement views among Chinese older adults. Additionally, the average educational level among the older population is relatively low, which could influence their capacity and methods for providing economic support.

## 5 Regression analysis of intergenerational economic support on older adults’ health

### 5.1 Regression results

The regression results, as shown in Table 2, indicate the impact of intergenerational economic support on the health status of older adults. Model 1, which includes all control variables, demonstrates that the majority of these variables significantly affect the health of older adults. Specifically, gender, age, the number of living children, and the number of grandchildren have a negative impact, whereas income, co-residence with children, and satisfaction with child relationships positively influence older adults’ health. Model 2 incorporates intergenerational economic support into the analysis and shows that the coefficient for intergenerational economic support is positive and significant at the 5% level. This suggests that providing financial support to children significantly improves the health of older adults, possibly because such support enhances their sense of being needed and valued, thereby positively affecting their physical and mental health.

**Table 2:** Regression analysis of intergenerational economic support on the health of the older adults.

Variables	Model 1	Model 2	Model 3
Intergenerational economic support		0.002^**^ (0.001)	0.021^***^ (0.004)
Gender	−0.170^***^ (0.016)	−0.170^***^ (0.016)	−0.171^***^ (0.017)
Age	−0.003^*^ (0.001)	−0.003^**^ (0.001)	−0.003^*^ (0.002)
Income	0.025^***^ (0.003)	0.024^***^ (0.003)	0.014^***^ (0.003)
Number of surviving children	−0.022^***^ (0.008)	−0.022^***^ (0.008)	−0.014 (0.009)
Number of grandchildren	−0.011^***^ (0.004)	−0.010^***^ (0.004)	−0.007 (0.004)
Co-residence with children	0.030 (0.022)	0.032 (0.022)	0.053^**^ (0.024)
Satisfaction with children	0.223^***^ (0.030)	0.225^***^ (0.030)	0.236^***^ (0.031)
Constant	−0.031 (0.096)	−0.034 (0.096)	−0.066 (0.102)
R^2^	0.074	0.075	
Instrument unavailability test			117.552^***^
Weak instrument test			111.104
Over-identification (*p*-value)			0.840
Estimation method	OLS	OLS	2SLS

*, **, ***indicate significance at the 10, 5, 1% levels.

To address potential endogeneity in the analysis, Model 3 employs the Two-Stage Least Squares (2SLS) method with robust standard errors. The validity of the instrumental variables is confirmed through a series of tests: the underidentification test (*p*-value <0.01), weak instrument test (F-statistic 111.104, exceeding the 10% critical value of 19.93), and overidentification test (*p*-value 0.840, indicating no overidentification issues), affirming the reliability of the instruments used. The regression coefficient for intergenerational economic support in Model 3 is 0.021, which is highly robust at the 1% significance level. This finding underscores the critical role that moderate economic support plays in enhancing the health of older adults and suggests that there may be an optimal level of financial assistance that maximizes health benefits. Comparing the OLS results from Model 2 with those from Model 3 indicates that the OLS may underestimate the positive effects of intergenerational economic support on older adults’ health, although the overall conclusions are consistent. For instance, with every 1% increase in the amount of economic support provided, there is a corresponding 0.021% improvement in the health status of older adults.

### 5.2 Threshold regression results

To explore whether the impact of intergenerational economic support on the health status of older adults varies across different threshold intervals, we conducted a threshold regression analysis based on the initial study. The results, presented in Table 3, reveal significant threshold effects. The test for a single threshold yielded a *p*-value of 0.000, significant at the 1% level; the double threshold showed a *p*-value of 0.033, significant at the 5% level; and the triple threshold had a *p*-value of 0.103, not significant. Consequently, the double threshold model was selected for empirical analysis.

**Table 3:** Threshold effect test.

Model	*F*-value	*p*-value	Bootstrap (BS)	Critical values		
				1%	5%	10%
Single	19.865^***^	0.000	300	6.386	4.397	3.317
Double	3.924^**^	0.033	300	6.089	2.283	0.255
Triple	2.994	0.103	300	9.211	4.432	3.190

*, **, ***indicate significance at the 10, 5, 1% levels.

Threshold estimates and confidence intervals obtained in the double threshold model, as shown in Table 4, indicate a single threshold value of 0.060 within a 95% confidence interval of [0.008, 56.500], and a double threshold value of 20.000 within a 95% confidence interval of [15.000, 21.000]. Based on these thresholds, the older adults in the sample (*N* = 5,137) were categorized into three groups: minimal intergenerational economic support (≤0.060), moderate support (0.060 < support ≤20.000), and substantial support (>20.000). Further exploration of the effects within these threshold intervals on older adults’ health is discussed below based on Table 5.

**Table 4:** Estimated threshold values and confidence intervals.

Model	Estimated value	95% Confidence interval
Single threshold	0.060	[0.008,56.500]
Double threshold	20.000	[15.000,21.000]

**Table 5:** Cross-sectional threshold model regression results.

Variable	Low IGS (IGS ≤ 0.060)	Moderate IGS (0.060 < IGS ≤ 20.000)	High IGS (IGS > 20.000)
Inter-generational economic support (IGS)	−4.105^**^ (1.688)	0.013^***^ (0.003)	0.000 (0.002)
Constant	−0.025 (0.132)	−0.078 (0.148)	0.029 (0.438)
Control variables	Control		
R^2^	0.076	0.076	0.109
N	2,862	2045	230

*, **, ***indicate significance at the 10, 5, 1% levels.

The results illustrate a significant nonlinear relationship, termed the “double threshold effect,” in the impact of intergenerational economic support on older adults’ health. When economic support is below the threshold of 0.060, the coefficient is −4.105, significant at the 5% level, suggesting that minimal economic support (less than 60 units of currency) adversely affects older adults’ health. This could be due to minimal interaction and emotional exchange between these older adults and their children, as well as poorer economic conditions and less investment in health, compounded by the decline in physical functions with age.

For support levels between 0.060 and 20.000, the coefficient is 0.013, significant at the 1% level, indicating that moderate levels of economic support positively impact older adults’ health. This support likely enhances their sense of self-worth and strengthens emotional bonds within the family, which are crucial for both mental and physical health.

Conversely, when economic support exceeds the threshold of 20.000, the effect on health is not significant, suggesting that excessive financial support does not improve the health of older adults. This scenario likely reflects the limited earning capacity of older adults, where excessive support could strain their finances and health, as the high demands of providing substantial support exceed their physical capabilities. Thus, while economic support can be beneficial, there is an optimal range beyond which no additional health benefits are observed.

### 5.3 Robustness checks

To verify the robustness of the main regression results, this section introduces tests that exclude certain age groups and employ alternative dependent variables for robustness checks.

#### 5.3.1 Excluding younger age samples

As individuals age, physical capabilities generally decline, meaning that older adults in the lower age range (60–69 years) might still retain better physical and labor capabilities compared to their older counterparts. To test the robustness of our findings, we reanalyzed the data excluding this younger cohort. The results, presented in Table 6, confirm that intergenerational economic support significantly and positively affects the health of older adults at a 5% significance level, with the direction and strength of control variables consistent with previous findings.

**Table 6:** Robustness results.

Variable	Excluding low-age samples (1)	Substituting dependent variable (2)
Intergenerational economic support	0.015^**^ (0.006)	0.283^***^ (0.056)
Control variables	Control	
R^2^	0.043	.
N	2,128	3,690

*, **, ***indicate significance at the 10, 5, 1% levels.

#### 5.3.2 Replacing the dependent variable

To further validate the reliability of our results, we employed the Physical Function Disability Scale as an alternative dependent variable. This scale provides an objective assessment of sensory and motor functions, crucial for gaging the physical quality of older adults. The regression results, also detailed in Table 6, remain consistent with the main findings, underscoring the robustness of the empirical outcomes.

### 5.4 Mediation effect analysis

According to Table 7, the first column shows that the total effect coefficient of intergenerational economic support on older adults’ overall health is positive and significant at the 5% level. The second column indicates that the regression coefficient of intergenerational economic support on older adults’ subjective life expectancy is positive. Concurrently, the third column reveals that the estimated coefficient of economic intergenerational support on older adults’ health status is significantly positive, suggesting an indirect effect. Additionally, in the third column, the coefficient of subjective life expectancy on older adults’ overall health status is significantly positive at the 1% level, indicating that economic intergenerational support also has a significant direct effect on older adults’ health. All regression coefficients being positive indicate that subjective life expectancy partly mediates the relationship between economic intergenerational support and older adults’ health status. This may be because as older adults provide economic support to their children, they find their place in their later years and can still realize their worth through their labor, fostering a positive and healthy aging perspective. This uplifting philosophy of life also enhances older adults’ subjective life expectancy. Moreover, an increase in subjective life expectancy suggests satisfaction with their health status and contentment with life in older age, which positive emotions can beneficially influence older adults’ health status.

**Table 7:** Mediation effect results of subjective life expectancy.

Variable	General health	Subjective life expectancy	General health
Intergenerational economic support	0.002^**^ (0.001)	0.002^**^ (0.001)	0.001^*^ (0.001)
Subjective life expectancy			0.395^***^ (0.016)
Control variables	Control		
R^2^	0.075	0.093	0.173
N	5,137	5,137	5,137

Standard errors in parentheses. *p < 0.05, **p < 0.01, **p < 0.001.

## 6 Conclusion and implications

In recent years, with the transformation of social structures and family patterns in China, intergenerational economic support between older adults and their children has emerged as a significant factor influencing elder health. This study challenges the traditional view that intergenerational support flows unidirectionally from children to parents. Our analysis reveals that by providing economic support to their children, older adults actively enhance their own health and quality of life, demonstrating that intergenerational support within families is a bidirectional and dynamic interaction. In China, even in later life, parents continue to support their children through household chores, grandparenting, and financial contributions, thereby maintaining their productivity (32). Older adults gain a sense of self-worth through supporting their children and respect through receiving support from them (33). In the context of traditional Chinese family culture, children are tasked with the responsibility of maintaining the health and care of the older adults, while parents also play a longstanding role in caring for their children. Thus, the impact of downward intergenerational support on health may often be overlooked. This paper, utilizing data from the 2018 China Health and Retirement Longitudinal Study and employing instrumental variable and threshold regression models, empirically analyzes the relationship between intergenerational economic support and older adults’ health, while also examining the nonlinear impact of intergenerational economic support across different threshold ranges. By incorporating a mediation effect model, the study further explores how subjective life expectancy influences the relationship between intergenerational economic support and older adult health.

Key findings include: First, there is a positive link between the economic support older adults provide to their children and their own health status. This link remains stable even after excluding younger older adult samples, indicating its broad applicability. This aligns with previous research suggesting a positive correlation between intergenerational support and older adults’ subjective well-being (34, 35). Li found that providing intergenerational support has a significant positive effect on perceived health. Additionally, scholars have found from the perspective of older adults’ life satisfaction that financial transfers to children correlate positively with the life satisfaction of older adults in China (36). Previous studies have mostly examined aspects like happiness, life satisfaction, and mental health among the older adults. This paper’s comprehensive indicators of perceived health, physiological health, and mental health extend and support reported results between downward intergenerational economic support and elder health, emphasizing its significance. Second, the study uncovers a dual-threshold effect of intergenerational economic support. Notably, moderate financial support from older adult parents to their children significantly enhances their health level, whereas excessive economic support has no significant effect (37). It is clear that providing financial support correlates positively with older adult health. However, economic pressure is a strong predictor of older adult health. Excessive financial support may indicate overwhelming economic pressure and overwork for the older adults, thus not significantly benefiting their health. This finding suggests that a balance must be found in the provision of intergenerational support, which is crucial for understanding how older adults can support their children without compromising their own quality of life. Third, subjective life expectancy plays a partial mediating role in the relationship between intergenerational economic support and older adults health, with significant mediation effects. This discovery emphasizes how older adults’ expectations for the future influence their daily behaviors and health status. Social support theory posits that intergenerational support benefits older adults’ capabilities and psychological resilience mechanisms (38). In China, older parents are reluctant to receive excessive economic support from their children, fearing it may burden them (39). Instead, they continue to provide significant support for their children and families, thereby gaining self-esteem, affirming their self-worth, and positively influencing their subjective life expectancy.

This paper’s findings offer several insights: First, providing a certain amount of financial support helps promote the physical and mental health of older adults, but excessive financial burdens can bring stress and adverse effects. Thus, it is crucial to find a sustainable support model to ensure that older adults’ health and welfare are not compromised by undue economic burdens. Employment can enable older adults to earn an income, realize their self-worth, and achieve mental fulfillment, which is beneficial for their health (40, 41). National and social organizations should provide opportunities for older adults to re-engage in the workforce, helping them realize their self-worth through work and ensuring they receive appropriate economic support at different life stages. Policies should also be developed to protect the rights of older adults in the workforce, providing them with employment and entrepreneurial subsidies. Second, the social support system for the older adults should be improved to reduce their economic burdens. Strengthening social security for the older adults, such as health insurance and pensions, can help reduce the risk of poverty due to illness. Additionally, the government should implement targeted economic assistance programs for the older adults, such as housing subsidies and living allowances, to ensure their quality of life post-retirement. Lastly, a scientific approach to intergenerational support should be advocated. Through education and advocacy, traditional societal views of the older adults should be changed, recognizing that older adults can still contribute meaningfully to society and their families in appropriate ways. At the same time, children’s financial support to the older adults should be moderate, avoiding demands that exceed the older adults’ capacity, thus protecting their self-esteem and self-efficacy, and promoting their health.

While this study robustly argues for the relationship between downward intergenerational economic support and older adult health, it still has some limitations. First, the use of cross-sectional threshold effects may overlook the average effects caused by temporal development, only estimating short-term effects. Future research could utilize panel data or longitudinal study designs to track individual changes over time for a more accurate understanding of these dynamics. Second, while this study uses samples from various regions in China, it cannot fully represent all areas, especially those in remote regions. Future studies could consider urban–rural differences, economic levels, and social class disparities. Lastly, this study explored the role of subjective life expectancy as a mediating variable, but there may be multiple mediating pathways between intergenerational support and older adult health. Future research could consider other potential mediators such as psychological stress, quality of life, and social participation.

## Data availability statement

To ensure transparency and reproducibility of this study, the primary data relied upon was sourced from the China Health and Retirement Longitudinal Study (CHARLS). Researchers interested in further investigation can access this data by visiting the official CHARLS website and adhering to the relevant data usage protocols. Access to the data requires registration and application through their website. For more information about CHARLS data, please refer to: https://charls.pku.edu.cn/.

## Ethics statement

The studies involving humans were approved by the Institutional Review Board (IRB) at Peking University. The studies were conducted in accordance with the local legislation and institutional requirements. The participants provided their written informed consent to participate in this study.

## Author contributions

LY: Conceptualization, Methodology, Writing – review & editing. ZX: Formal analysis, Methodology, Writing – original draft, Writing – review & editing.
